# Associations between migraine and possible risk factors in the Czech Republic

**DOI:** 10.3389/fneur.2023.1256650

**Published:** 2023-09-26

**Authors:** Petra Riedlova, Barbora Zahradnikova, Dagmar Skybova, Hana Slachtova, Vitezslav Jirik, Hana Tomaskova

**Affiliations:** ^1^Centre of Epidemiological Research, Faculty of Medicine, University of Ostrava, Ostrava, Czechia; ^2^Department of Epidemiology and Public Health, Faculty of Medicine, University of Ostrava, Ostrava, Czechia

**Keywords:** risk factors, migraine, air pollution, anxiety, depression

## Abstract

**Introduction:**

Migraine is a widespread neurological disorder, growing increasingly common. However, the pathogenesis of the disease is often unclear and the evidence for the role of various risk factors is limited. This study aimed to identify risk factors associated with migraine and to contribute towards a better understanding of this disease.

**Methods:**

Data from 3,247 questionnaires were analyzed for associations between migraine and sex, age, BMI, degree of education, and air pollution, along with other factors such as contact with friends, physical condition, health, anxiety, and depression. A cross-sectional study was conducted with an approximately equal distribution of the sample by age, gender and two analysed regions. Data were presented using basic descriptive statistics using the chi-square test. The model output was presented using a crude odds ratio (OR) and a fully adjusted OR. Three hundred and eight-six individuals (12%) suffered from migraine.

**Results:**

In an adjusted model, the presented study found associations between the prevalence of migraine and sex, age, and level of education. Individuals with migraine statistically significantly more often suffered from depression, anxiety and other selected factors. However, the assumed significant association between the occurrence of migraine and pollution in the region has not been found

## 1. Introduction

Migraine is a widespread neurological disorder, most commonly associated with episodic attacks of headaches. These headaches are often accompanied by other neurological symptoms, such as various types of aura or transient neurological deficit (1). Migraine does not threaten the life of the patient, but it can significantly reduce the quality of life and even prevent the patient from normal daily activities. Migraine is increasingly common, with the possible involvement of many risk factors (RF) including, for example, air pollution, gender, or even education (1, 2). In most patients, however, the pathogenesis of the disease is unclear and the evidence for the role of various risk factors is limited (1, 2).

In the past, associations between migraine, sex, age, BMI, degree of education, and air pollution have been investigated (1, 3–18), along with other factors such as contact with friends, physical condition, health, anxiety, and depressions (18–27). The factors mentioned above are also addressed in the present study.

Worldwide, more than 1 billion people suffer from migraine (28). The prevalence is highest in Europe and North America (12.6–14.7%), approx. 18% in women and approx. 6.5–8% in men, respectively. The lowest prevalence is, on the other hand, in African and Asian countries (29). The prevalence of migraine in the Czech Republic has been estimated at approx 16% of the population (30). Patients suffering from migraine often report the environment to be a trigger for their headaches – this may include a change in the atmospheric pressure, bright sunlight, air quality, or various odors. The environmental aspects play a role also in the course of individual attacks (for example, a dark room may improve the headache) (31). As migraine has a major socioeconomic impact on the population, mitigating environmental triggers that could possibly help in preventing attacks of migraine is an important research topic (31).

This study aimed to identify risk factors associated with migraine and to contribute towards a better understanding of this disease.

## 2. Methods

### 2.1. Description of the participant group

**Figure 1:**
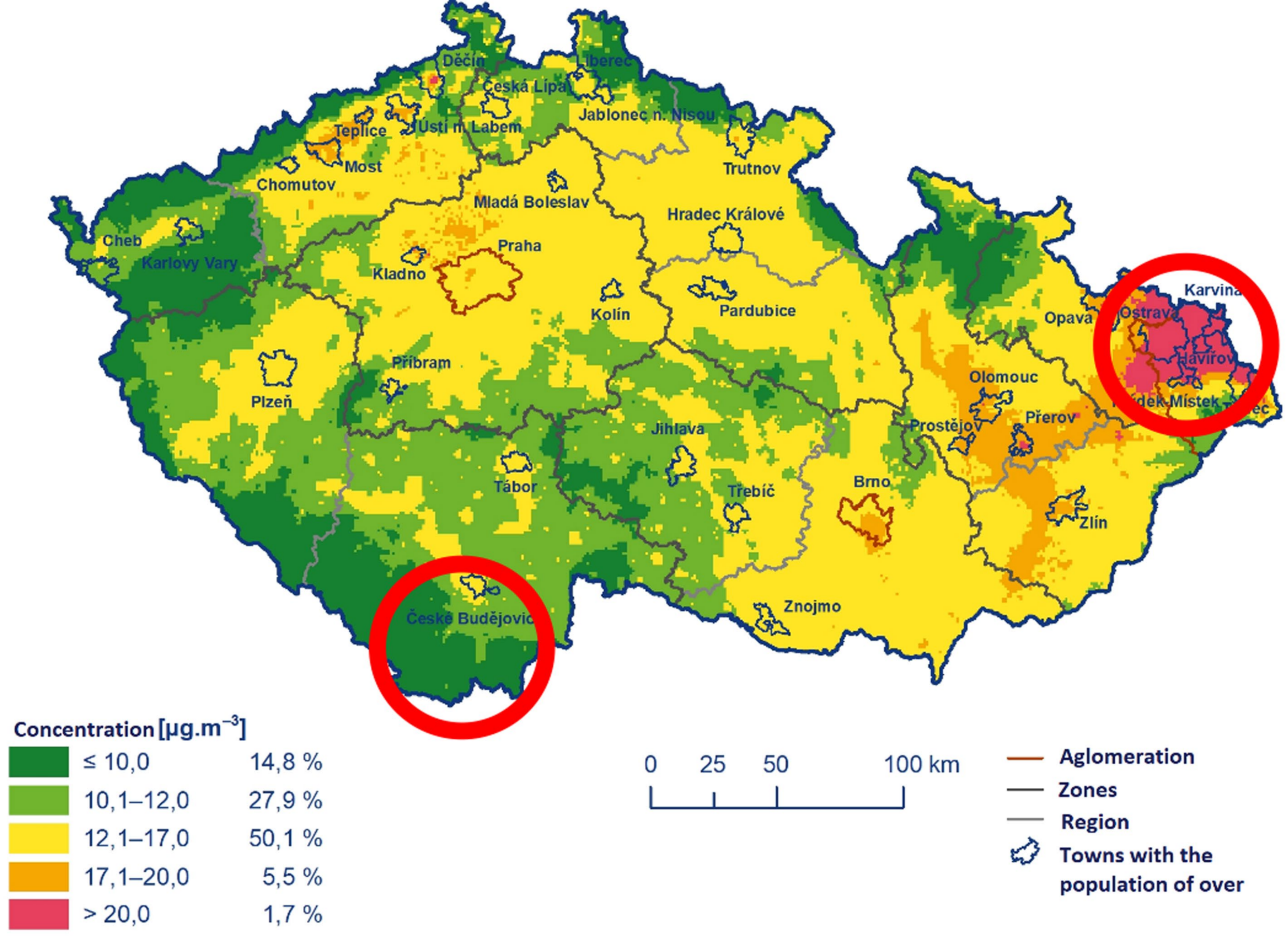
Five-year average of annual mean PM2.5 concentrations (2016–2020).

Three thousand two hundred and forty-seven questionnaires filled in 2022 by participants programme 1 (the middle-aged cohort) of the project Healthy Aging in Industrial Environment (HAIE, a project evaluating the effects of environmental pollution on various aspects of human health) were included in the study. The questionnaire has been approved by the Ethics Committee University of Ostrava (nr. 2/2018) and respondents signed informed consent. The questionnaires included among other things the data about the place of residence of the participants, their age (categories 35–40, 41–45, 46–50, 51–55. 56–60, 61–65, and sex as well as a wide range of diseases and health problems) (32). The inclusion criteria were: the participant’s consent with being included in the study, age of 35–65 years, living in one of the two studied regions of the Czech Republic (Moravian-Silesian region, MSR – industrial area, the 5-year average annual concentration of PM_2,5_ was more than 20 μg/m3 and the average annual concentration of PM_10_ was up to 30 μg/m3 and South Bohemian region, SBR, non-industrial area, the five-year average of annual concentration of PM_2,5_ was less than 12 μg/m3 and the average annual concentration of PM_10_ was up to 20 μg/m3) (15, 32, 33) (Figure 1). These persons have lived in these locations for at least half of their lives, including at least 5 years during childhood (i.e., before the age of 15) and at least the last 10 years.

### 2.2. Design of the study

It is a cross-sectional study. The design of the study supported an approximately even distribution of the population according to the principal parameters (age, sex) between the regions. The participants provided subjective evaluation in the questionnaire (32). The presented study utilizing these questionnaires primarily focused on migraine and its association with potential risk factors (sex, age, BMI, degree of education, and air pollution have been investigated along with other factors such as contact with friends, physical condition, health, anxiety, and depressions). Physical condition and subjective perception of health were classified on a scale of 1–5 (1 – best; 5 – very poor), age was used as an ordinal variable, and the remaining factors were considered binary (present-absent).

### 2.3. Statistical analysis

The data are presented using basic descriptive statistical parameters (arithmetic mean, standard deviation, frequency tables). Differences in the occurrence of the individual factors between groups with and without migraine were tested using the chi-square test. The association was also evaluated using binary logistic regression including the variables of the region (MSR, SBR), sex, age, BMI, and education.

The model output was presented using a crude odds ratio (OR) and a fully adjusted OR with 95% confidence intervals (CI). The level of significance was set 5%. All analyses were performed in Stata version 17.

## 3. Results

### 3.1. Group characteristics

**Figure 2:**
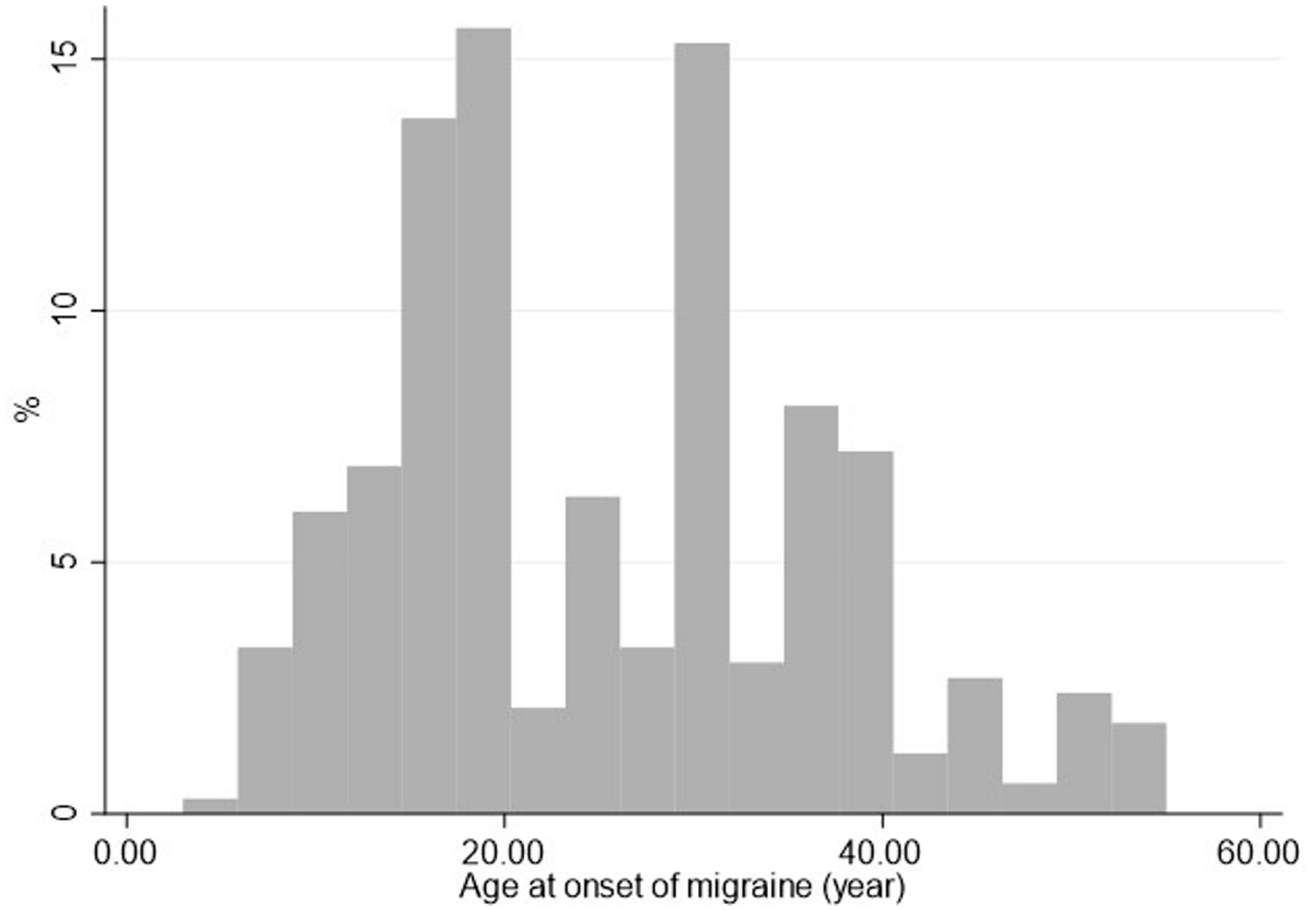
Distribution of the age of onset of migraine.

In all, 3,247 individuals participated in the study, with a mean age of 48.1 ± 7.6 years in the SBR cohort (*n* = 1,242 participants, 38.3%) and 49.2 ± 8.0 years in the MSK cohort (*n* = 2,005 participants, 61.7%), respectively. Three hundred and eighty-six individuals (12%) suffered from migraine; the mean age at diagnosis was 25.3 years of age (Figure 2). More than half (52%) are diagnosed by age 25. After the age of 45, it is about 5%. The mean period between the onset of migraine and filling out the questionnaire was 21.9 years.

### 3.2. Associations between risk factors and migraine

The prevalence of migraine did not significantly differ between the regions with different pollution levels (*p* = 0.880). As obvious from Table 1, women suffer from migraine significantly more often than men (16% vs. 5%; *p* < 0.001). The prevalence of migraine also declined with the age of participants at the time of the study (*p* = 0.002). BMI was also associated with the prevalence of migraine, with the highest prevalence being observed in the group with normal BMI (15%) and the lowest prevalence in obese persons (9%). Where education is concerned, the prevalence in individuals with only lower secondary/vocational education was significantly lower than in those with secondary or university education (*p* < 0.001). The data are summarized in Table 1.

**Table 1:** Risk factors significantly associated with migraines.

		Migraines present	Migraines absent	*P*-value*
		*n* (%)	*n* (%)	
Region	SBR (*n* = 1,242)	149 (12%)	1,093 (88%)	0.880
	MSR (*n* = 2005)	237 (12%)	1,768 (88%)	
Sex	Men (*n* = 1,290)	65 (5%)	1,225 (95%)	<0.001
	Women (*n* = 1935)	318 (16%)	1,617 (84%)	
Age	35–39 (*n* = 415)	65 (16%)	350 (84%)	0.002
	40–44 (*n* = 697)	92 (13%)	605 (87%)	
	45–49 (*n* = 727)	99 (14%)	628 (86%)	
	50–54 (*n* = 544)	52 (10%)	492 (90%)	
	55–59 (*n* = 477)	44 (9%)	433 (91%)	
	60–65 (*n* = 387)	34 (9%)	353 (91%)	
BMI	16–25.0 (*n* = 1,115)	164 (15%)	951 (85%)	0.001
	25.1–30.0 (*n* = 1,259)	143 (11%)	1,116 (89%)	
	>30.0 (*n* = 836)	76 (9%)	760 (91%)	
Education	Lower secondary/Vocational (*n* = 886)	68 (8%)	818 (92%)	<0.001
	Higher secondary (*n* = 1,454)	195 (13%)	1,259 (87%)	
	Higher/University (*n* = 903)	123 (14%)	780 (86%)	

*Chi^2^-test.

### 3.3. Adjusted model

The adjusted model revealed no significant difference in the risk of migraine development between the polluted and unpolluted region (Odds Ratio OR = 1.16; 95% CI 0.92–1.45). Sex was the most significant risk factor, with the risk of developing migraine approx. 3.5 times higher in women than in men. The risk declined with age, with the age group >50 years of age having a significantly lower chance of suffering from migraine than those in the age group 35–39 years of age. Unlike in unadjusted analysis, no statistically significant differences were found with respect to BMI. Individuals with higher/university education had statistically significantly increased odds of suffering from migraine than those with only lower secondary/vocational education, see more in Table 2.

**Table 2:** Adjusted model of calculation of the selected risk factors.

Variable	Category	Crude OR (95% CI)		Adjusted OR (95% CI)		*P*-value
Region	SBR (*n* = 1,242)	ref.				
	MSR (*n* = 2005)	0.98	(0.79–1.22)	1.16	(0.92–1.45)	0.213
Sex	Men (*n* = 1,290)	ref.				
	Women (*n* = 1935)	3.71	(2.81–4.89)	3.54	(2.66–4.71)	<0.001
Age	35–39 (*n* = 415)					
	40–44 (*n* = 697)	0.82	(0.58–1.15)	0.77	(0.54–1.10)	0.147
	45–49 (*n* = 727)	0.85	(0.60–1.19)	0.85	(0.60–1.21)	0.366
	50–54 (*n* = 544)	0.57	(0.39–0.84)	0.57	(0.38–0.86)	0.007
	55–59 (*n* = 477)	0.55	(0.36–0.82)	0.55	(0.36–0.83)	0.005
	60–65 (*n* = 387)	0.52	(0.33–0.81)	0.51	(0.32–0.81)	0.004
BMI	16–25.0 (*n* = 1,115)	ref.				
	25.1–30.0 (*n* = 1,259)	0.74	(0.58–0.94)	1.02	(0.79–1.32)	0.868
	>30.0 (*n* = 836)	0.58	(0.43–0.77)	0.81	(0.60–1.09)	0.165
Education	Lower secondary/Vocational (*n* = 886)	ref.				
	Higher secondary (*n* = 1,454)	1.86	(1.39–2.49)	1.57	(1.16–2.12)	0.003
	Higher/University (*n* = 903)	1.90	(1.39–2.59)	1.6	(1.16–2.22)	0.004

ref., reference category; OR, odds ratio; SBR, region of South Bohemia; MSR, Moravian-Silesian Region.

Last but not least, the study focused on the influence of migraine on the quality of life. Individuals with migraine statistically significantly more often (*p* < 0.001) suffered from depression (17% vs. 5%), anxiety (30% vs. 10%), poorer health (9% vs. 5%), poorer physical condition (14% vs. 23%) and their contact with friends was more-often limited (31% vs. 23%). The association of these factors was significant after adjustment to age and sex as well, see Table 3.

**Table 3:** Selected factors and their associations with the presence of migraines after adjustment for sex and age.

		Individuals	Migraines			Adjusted OR (95% CI)*		
		*n* (%)	Absent (%)	Present (%)	*P*-value			*P*-value
Depression	No	3,029 (93%)	2,708 (95%)	321 (83%)	<0.001			
	Yes	218 (7%)	153 (5%)	65 (17%)		3.16	(2.29–4.36)	<0.001
Anxiety	No	2,845 (88%)	2,573 (90%)	272 (70%)	<0.001			
	Yes	402 (12%)	288 (10%)	114 (30%)		3.27	(2.52–4.23)	<0.001
General health	1	332 (10%)	309 (11%)	23 (6%)	<0.001	ref.		
	2	1,581 (49%)	1,398 (49%)	183 (48%)		2.07	(1.31–3.27)	0.002
	3	1,131 (35%)	988 (35%)	143 (37%)		2.43	(1.52–3.88)	<0.001
	4 + 5	188 (6%)	152 (5%)	36 (9%)		4.10	(2.3–7.33)	<0.001
Physical condition	1	210 (6%)	195 (7%)	15 (4%)	<0.001	Ref.		
	2	1,027 (32%)	923 (32%)	104 (27%)		1.53	(0.86–2.7)	0.146
	3	1,520 (47%)	1,341 (47%)	179 (46%)		1.67	(0.96–2.91)	0.071
	4 + 5	478 (15%)	391 (14%)	87 (23%)		2.67	(1.49–4.8)	0.001
Frequent contact with friends	No	764 (24%)	645 (23%)	119 (31%)	<0.001	1.70	(1.34–2.16)	<0.001
	Yes	2,470 (76%)	2,204 (77%)	266 (69%)				

*After adjustment to age and sex.

## 4. Discussion

This study aimed to identify risk factors associated with migraine and to contribute to the understanding of this disease, which could potentially help in the prevention of its development.

In all, 11.9% of study participants suffered from migraine. This result is consistent with those reported elsewhere both for the Czech Republic and other countries (29, 30, 34, 35). The mean duration of the disease was almost 22 years (but note this is not representative of the general population as only respondents aged 35–65 years were included in our study).

The fact that women suffer from this disease more commonly than men (16% vs. 5%, respectively), is in accordance with data published elsewhere (5, 12, 16, 34). This is, as discussed below in more detail, likely associated with sex hormones. This difference was significant even in the adjusted model (Table 2), with a 3.5 times higher risk of developing migraine in women than in men.

Surprisingly, no significant relationship between living in the polluted (industrial area) or unpolluted region (non industrial area) and the migraine prevalence was observed, nor even after adjustment (Table 2). Although the risk was slightly higher in the polluted region (OR = 1.16), this difference was not statistically significant. This result is another piece in the mosaic of studies that have tackled this problem. However, most studies have examined the short-term effects of air pollution in relation to migraine (1, 2, 11, 16, 36, 37), which cannot be compared with our long-term exposures results. Data on long-term exposures in relation to migraine are very scarce. However, they suggest that chronic exposure may be important in the etiopathogenesis of migraine (38).

However, these differences can be affected by many other factors, such as the general lifestyle, stress, or the particular type of pollution. This stress may have synergic effects with pollution and, in effect, inflate the significance of pollution as a risk factor. The size of the cities and towns in the study areas may be another factor influencing the results as the aforementioned studies that found differences between polluted and unpolluted areas were performed in big cities in Chile, Korea, Taiwan, or Canada. Although the MSR is generally considered to be one of the most polluted areas in the Czech Republic, cities here are much smaller than on other continents (to compare, the largest city in the heavily polluted region, Ostrava, has a population of approx. 300,000, all other cities/towns are well below 100,000) (39). As mentioned above, the type of pollution can also have an impact. If there is, for example, a metallurgical or heavy engineering industry in the cities, this may play a greater role than the traffic associated with the size of the city. In our study, the metallurgical and engineering industries are located in the polluted region (MSR) compared to the unpolluted region (SBR), where these industries are absent (40). We must also consider the climate as some studies have shown that very damp or, on the other hand, arid climate can also increase the risk of migraine (1, 2, 16, 36, 41). Considering only Europe, some regions in Poland, Italy, and Croatia are counted among the most polluted regions (42).

Our results also imply that age was a significant risk factor, higher age on filling out the questionnaire was associated with a lower prevalence of migraine. This might be caused by the fact that women suffer from migraine more often than men. As the level of female sex hormones gradually decreases in older age and migraine are often associated with the menstrual cycle, it is logical that after menopause, when the level of sex hormones stabilizes, the occurrence of migraine also dwindleeuros (4, 13, 14). This is obvious from data in Table 1 showing that the incidence of migraine sharply decreases after about the 50th year of age. This effect was observed after adjustment as well (Figure 2; Table 2).

Normal BMI appeared to be a risk factor when evaluating the direct unadjusted association between this parameter and migraine (Table 1). After adjustment, however, the effect was no longer significant (Table 2). This phenomenon can be explained by the high association between sex and migraine – women are more commonly affected by migraine than men and at the same time, they are more BMI-conscious and tend towards a more healthy lifestyle, which makes them more likely to have normal BMI. On the other hand, Miri et al. (9) and Yu et al. (17) reported an association between higher BMI (>30) and migraine, which could be associated with the general lack of physical activity in such people.

The last evaluated possible risk factor, the achieved level of education, revealed a significantly reduced prevalence of migraine among those with the lowest education (Table 1). After adjustment, individuals with higher degrees of education had approx. 1.5 times higher risk of suffering from migraine than those with only lower secondary or vocational education. This could be indirectly caused by greater stress – higher education level is associated with higher responsibilities and, in effect, stress, which could also cause migraine. This has been also reported by Queiroz et al. (43). On the other hand, studies by Han Le et al., Atasay et al., and Moon et al. found the lower education to be associated with a higher prevalence of migraine. The explanation in these papers lies in generally higher demands on physical activity of such individuals as they have to labor physically, which could also be a triggering cause for migraine. An unhealthy lifestyle in individuals with lower education level can be also considered an important factor (3, 6, 10).

The relationship between migraine and other selected diseases (anxiety and depression) detected in our study is also interesting (Table 3). This result corroborates those reported by other studies (19, 20, 22–24, 26, 27). Luo et al. analyzed the association between migraine and anxiety in middle-aged women and found a 1.28 times higher risk of developing anxiety in women suffering from migraine than in those without this disease (22). In our study, this risk is even higher (migraine is associated with more than three times higher risk of anxiety). This can be caused by different populations – while our study is in a Central European population, the study by Luo et al. was conducted in the United States; it is possible that the cultural differences such as a possibly higher degree of stress in either of the countries may be the reason for this difference.

In the American population, Buse et al. found migraine to be associated with a two-fold increase in the risk of developing depression or anxiety (19). Ten years later, the same authors showed that individuals from the cohort of patients with migraine had a 2–3 times higher risk of sleeping disorders, depression, anxiety, or gastric ulcers (44).

A statistically significant association was also found between migraine and general health, physical condition, and contact with friends. Individuals suffering from migraine generally reported lower quality of life (worse health, worse physical condition, worse social life), which is in line with previous studies (21, 25). Migraine negatively affect both social life and physical fitness. This fact is confirmed by a study conducted on 300 people between 20 and 54 years old by Al Harbi et al. (45). Similarly, a review study that compared 80 studies concluded that migraine greatly affects overall quality of life (46). It must be, however, noted that these are associations and nothing could be inferred about the causality – it has not been studied whether migraine preceded the lower quality of life or whether the lower quality of life was the cause for developing migraine (21). We are rather inclined to think that it is the migraine that cause anxiety and depression as this problem can influence the well-being to such a degree that a patient suffering from frequent migraine might be virtually debilitated and migraine can prevent him/her from social life or sports. In our opinion, this is a much more likely causative link than the other possibility. However, in western countries, it is generally reported that, regardless of geographic location, migraineurs are more likely to be female, educated, have a normal BMI and an anxious-depressed baseline personality (47).

The character of the data from the questionnaires can be considered a limitation of this study as they were self-reported and, therefore, inherently subjective (although data on medical diagnoses were verified). The absence of young individuals under 34 years of age can be also considered a limitation that prevented us from exploring the entire age range. The exposures themselves may have been a limitation. In this case, we are based on average annual exposures; we are not assessing individual exposures. It is, of course, likely that some factors that might have influenced the development of migraine have not been captured in the questionnaire and additional questions could be beneficial for a more complex evaluation of the issue. For example, genetic factors, stress-related factors and microclimatic factors (humidity, heat, pressure). On the other hand, the even distribution of study participants with respect to sex, age, and region can be considered a strong suit of this study.

## 5. Conclusion

The study focused on whether living conditions and lifestyle can indirectly influence the prevalence of migraine. In an adjusted model, the presented study found associations between the prevalence of migraine and sex, age, and level of education. However, the assumed significant association between the occurrence of migraine and pollution in the region has not been found. We have also found a significant association between migraine and depression, anxiety, and limited social life. However, the possible relationship between migraine and air pollution should continue to be addressed.

## Data availability statement

The original contributions presented in the study are included in the article/Supplementary material, further inquiries can be directed to the corresponding author.

## Ethics statement

The studies involving humans were approved by Ethics Committee University of Ostrava (nr. 2/2018). The studies were conducted in accordance with the local legislation and institutional requirements. The participants provided their written informed consent to participate in this study.

## Author contributions

PR: Conceptualization, Investigation, Writing – original draft. BZ: Data curation, Writing – original draft. DS: Data curation, Writing – original draft. HS: Formal analysis, Methodology, Supervision, Writing – review & editing. VJ: Supervision, Writing – review & editing. HT: Formal analysis, Methodology, Supervision, Validation, Writing – review & editing.
