# Supplementary material for: Associations between migraine and possible risk factors in the Czech Republic
Source: Front Neurol. 2023 Sep 26;14:1256650. doi: 10.3389/fneur.2023.1256650 (PMC10562564; doi:10.3389/fneur.2023.1256650)
Supplement: Supplementary file 1 [file Data_Sheet_1.PDF]

Projekt Healthy Aging in Industrial
Environment HAIE
CZ.02.1.01/0.0/0.0/16_019/0000798

A Basic demographic characteristics

**ID of respondent**		**ID of interviewer**	

A1	Sex	1	man
		2	woman

A2	Your year of birth	

A7	Your highest educational attainment	1	Basic (and incomplete)
		2	Vocational without diploma
		3	High school diploma (general and vocational)
		4	Higher vocational (post-secondary)
		5	University and higher

C1 How do you assess your overall health?

C Health status

	1	very good
	2	good
	3	satisfactory
	4	poor
	5	very poor

C4	Do you suffer from any chronic diseases?		
		Yes	No

C6 Do you have or have you ever had any of the following diseases or health problems?

	C6.1	*<u>Indicate in the first column</u> ('presence of disease') in each row of the following table whether you have the disease or condition*				
	C6.2	*Indicate <u>in the second column</u> ('diagnosis by a doctor') in each row of the following table whether you have been diagnosed with the disease (if present) by a doctor*				
	C6.3	*Indicate <u>in the third column</u> ('limitations') in each row of the following table whether the illness or medical condition you have suffered from is a medical condition. (if any) restricts you in your daily life or sporting activities*				
	C6.4	*Indicate by the number <u>in the last column</u> how old you were when the disease or medical condition (if any) was diagnosed*				
			Presence of the disease	Diagnosis by a doctor	Limitations	How old were you when you were diagnosed
			C6.1	C6.2	C6.3	C6.4
	1	Asthma (including allergic asthma)				
	2	Chronic bronchitis, other chronic obstructive pulmonary disease, emphysema				
	3	Myocardial infarction (heart attack) or its chronic sequelae				
	4	Ischaemic heart disease (including angina pectoris)				
	5	High blood pressure (hypertension)				
	6	Elevated cholesterol levels				
	7	Stroke (brain haemorrhage, thrombosis of the cerebral arteries) or chronic sequelae of stroke				
	8	Osteoarthritis (non-inflammatory joint disease)				
	9	Inflammatory joint disease (rheumatoid arthritis)				
	10	Diseases of the lumbar spine and sacrum or other long-term back problems				

		C6 – continued	Presence of the disease	Diagnosis by a doctor	Limitations	How old were you when you were diagnosed
	11	Diseases of the cervical spine or other long-term problems in the cervical spine				
	12	Type II diabetes				
	13	Allergies (hay fever, eye infections, skin, food and other allergies - except allergic asthma)				
	14	Liver cirrhosis				
	15	Migraine and other severe headaches				
	16	Problems with urinary retention, problems with conscious bladder control				
	17	Kidney problems (severe, chronic)				
	18	Depression				
	19	Mental tension, anxiety				
	20	Thyroid problems				
	21	Osteoporosis				
	22	Other...				
	23	Cancer				
	24	Alzheimer's disease				
	25	Dementia (e.g. vascular, Parkinson's disease)				
	26	Immunodeficiency (immune system disorder)				

C13	How do you assess your physical condition?					
	Please indicate the number expressing your opinion	Very good	Good	Average	Not too good	Poor
		1	2	3	4	5

C14	Your height /in cm/	

C15	Your current weight /in kg/	
	If you are a pregnant woman or shortly after giving birth, indicate your pre-pregnancy weight.	

C16	Your waist circumference /in cm/	

D Lifestyle

D2	Describe your **physical activity (outside of work)** in the activities you do, e.g. working around the house, caring for your family, studying, etc.	
	1	predominantly sedentary activity or standing
	2	predominantly walking or moderate physical activity
	3	predominantly heavy work or physically demanding activity
	4	no physical work activity

D3	**During the past month**, how much time did you spend on physical activity - on average, how many times a week and for how long (at least 10 minutes) did you do the following types of physical activity on average? Please indicate only the types of physical activity you did in your free time, outside of work. Do not include activities that you do at home or at school. If you did not engage in any physical activity, the answer will be '0'. In the third column, indicate whether the physical activity was done outdoors (enter 1) or indoors - gym, swimming pool, etc. (enter 2)				
			How many times per week	Average duration of one activity (in minutes)	Outdoors or indoors
	a)	Strenuous physical activity (fast heart rate, sweating) - e.g. running, hockey, football, basketball, cross-country skiing, intense rollerblading, cycling, intense swimming			
	b)	Moderate physical activity (not strenuous, light sweating) - e.g. fast walking, light cycling, dancing, volleyball, badminton, light swimming, downhill skiing, intensive gardening			
	c)	Moderate physical activity (minimal effort, no sweating) - e.g. light walking, stretching, bowling, petanque, fishing, light gardening			
	d)	Time spent sitting - e.g. sitting at a table, visiting friends, reading, watching TV			

E Satisfaction with life/quality of life

E1	How would you rate your quality of life?	
	The quality of life assessment is an overall assessment of satisfaction with oneself with yourself and your life in all aspects, such as your living conditions and environment, work, leisure and daily activities, health, financial and economic situation, family and social background, personal relationships, etc.	
	1	very good
	2	good
	3	neither good nor bad
	4	bad
	5	very bad

E4	You would say that your health is generally:	
	1	Excellent
	2	Very good
	3	Good
	4	Decent
	5	Poor

E11	Do you have frequent contact with friends?	Yes	No

E15	How many of your friends would you say you have a close relationship with?	
	(write the number)	

This is the end of the questionnaire.
Thank you for completing it.
